# Persistent opioid use is associated with a shorter myeloma survival

**DOI:** 10.1007/s00520-025-09407-8

**Published:** 2025-04-11

**Authors:** Karen Sweiss, Ana Maria Avila Rodriguez, John G. Quigley, Lisa Sharp, Kaily Kurzweil, Jaleel G. Sweis, Gregory S. Calip, Zacharie Hamilton, Chukwuemeka Uzoka, Elizabeth Ilo, Elliot Wolf, Damiano Rondelli, Douglas W. Sborov, Pritesh Patel, Craig C. Hofmeister

**Affiliations:** 1 Department of Pharmacy Practice, College of Pharmacy, University of Illinois, 833 South Wood Street, Chicago, IL 60612 USA; 2 Division of Hematology/ Oncology, University of Illinois at Chicago, Chicago, IL USA; 3 Center for Pharmacoepidemiology and Pharmacoeconomic Research, University of Illinois at Chicago, Chicago, IL USA; 4 Bone Marrow Transplant and Cellular Therapy, Northwestern Memorial Hospital, Chicago, IL USA; 5 Division of Hematology and Hematologic Malignancies, Huntsman Cancer Institute at the University Salt, Lake City, UT USA; 6 Department of Hematology and Medical Oncology, Winship Cancer Institute of Emory University, Atlanta, GA USA

**Keywords:** Opioids, Pain, Multiple myeloma, Autologous transplant

## Abstract

**Purpose:**

Overuse of opioids has been associated with a significant public health crisis, yet remain critical as adjunctive treatment in multiple myeloma (MM). Questions remain about the balance between the benefits to risks of ongoing opioid use, especially when the patient’s myeloma is in remission. This retrospective review focuses on the association of chronic opioid use (COU) in a patient population predominantly people of color.

**Methods:**

A cohort of 174 MM patients who received autologous stem cell transplant (ASCT) at an urban medical center were studied.

**Results:**

Baseline COU was observed in 52.9% of patients. COU rates and average morphine milligram equivalents (MME) per day were similar in those with and without bone disease. Previous illicit drug use was associated with a higher baseline COU. 142 (81.6%) patients received opioids during ASCT admission, while 105 (60.3%) were discharged on opioids and 72 (41.4%) met criteria for COU at 6 months. Opioid use at hospital discharge was associated with a higher 6-month COU (p = 0.008). COU at 6 months was independently associated with worse overall survival (p = 0.006).

**Conclusion:**

We describe high rates of baseline COU in MM unrelated to bone disease. A number of patients started opioids during ASCT and were still taking them at 6-month follow-up visit. We demonstrate a negative association of 6-month COU on OS but not on PFS suggesting that opioid-related morbidity may play a role. These data highlight the need to test causality of opioid use on survival while simultaneously improving the management of pain in MM.

## Introduction

Osteolytic bone lesions are a frequent complication of multiple myeloma (MM) [1], increasing morbidity and mortality [2]. Patients may also develop peripheral neuropathy from thalidomide or bortezomib which decreases quality of life and can persist after completion of treatment [3]. Thus, opioids are often thought of as a cornerstone of myeloma treatment [4].

In recent years, driven by the opioid epidemic, increased attention has been given to opioid risks in the treatment of noncancer pain, and there has been an increase in guidelines and consensus recommendations aimed at addressing opioid overuse and/or misuse [5, 6]. However, the CDC guideline for prescribing opioids for chronic pain excludes patients undergoing active cancer treatment, even though similar levels of pain have been reported in patients during and after completion of cancer treatment [7]. Emerging data suggest that patients with cancer are at equal risk of using a prescription opioid in a way not directed by the prescriber when compared with patients without cancer [8], even though cancer patients are ten times less likely to die from opioid overdose versus the general population [9].

The majority of cancer survivors receive opioids during cancer treatment, and at least a third will continue to experience chronic pain even after completing cancer treatment [10]. While opioids are an important aspect of pain management during cancer treatment, their role for those who have completed curative treatment is less clear. Recent studies have demonstrated that cancer survivors are more likely to receive long-term and high dose opioid prescriptions compared to patients without cancer, particularly in the early years after their diagnosis [8, 11].

The consequences of long-term opioid use in patients with hematologic malignancies are not well described. While emergency department visits for opioid overdose in cancer patients is extremely rare, one report using the Healthcare Cost and Utilization Project Nationwide Emergency Department Sample of 35,339 opioid-related emergency department visits in cancer patients noted that MM patients were at increased risk for overdose (OR 1.73, 95% CI 1.32–2.26); interestingly almost none of those patients had myeloma bone disease, but a quarter were classified as having a mood or substance use disorder [12]. We hypothesized that MM patients with extensive myeloma bone disease or pathologic compression fractures would have the highest prevalence of opioid use, and that these patients would continue to use opioids whether they reached remission or not. In an unselected cohort from an academic center serving predominantly minority patients we aimed to quantify chronic opioid use before autologous stem cell transplant (ASCT), identify correlates (disease characteristics, sociodemographic or psychosocial) of chronic opioid use at baseline and at 6 months after transplant, and determine whether opioid exposure is associated with survival outcomes post-transplant.

## Methods and statistical analysis

### Study design

After obtaining institutional review board approval, we performed a single-center retrospective chart review of adult patients ≥ 18 years with a confirmed diagnosis of multiple myeloma using the International Myeloma Working Group (IMWG) and World Health Organization (WHO) criteria and who received high dose melphalan 140–200 mg/m^2^ followed by ASCT between January 1, 2005 and December 30, 2019.

### Data collection

Patient demographic, clinical, and treatment data were abstracted from our electronic medical record (EMR) and entered into a secure online Research Electronic Data Capture (REDCap) system [13]. Data regarding factors that have been previously associated with opioid use in noncancer patients such as age, gender, race, history of substance use disorder (i.e., smoking, alcohol, and illicit drugs), history of psychiatric disease (i.e., depression, anxiety, and other psychiatric illnesses), insurance status (i.e., Medicaid, Medicare, private, or other), employment status (i.e., unemployed, disability, employed, or retired), and education level (i.e., none, high school, GED, technical, or college degree) were collected. Disease and treatment-related factors were collected including the presence of bone disease, a history of skeletal-related events prior to ASCT (i.e., a pathologic fracture, spinal cord compression, necessity for radiation to bone for pain or impending fracture or surgery to bone), time to ASCT from diagnosis, myeloma protein subtype, number of prior lines of therapy, receipt of novel agent induction and/or post-ASCT maintenance therapy, pre- and post-ASCT IMWG response, the presence of high risk fluorescent in-situ hybridization (FISH) or karyotypic lesions [14], and International Staging System (ISS) at diagnosis. Bone disease in MM was defined as the presence of osteolytic bone lesions or the presence of osteoporosis with compression fractures attributable to the underlying clonal plasma cell disorder. Because this retrospective study included patients diagnosed with myeloma before and after the updated 2014 IMWG criteria were adopted, conventional skeletal radiography, MRI, FDG-PET, PET-CT or whole-body CT were all acceptable as imaging technologies to demonstrate bone disease [15].

#### Objectives

Our primary endpoint was persistent chronic opioid use (COU) 6 months following ASCT as all melphalan-related toxicities other than fatigue have resolved by 6 months following ASCT. COU was defined as having any prescribed opioids or dosing for at least 90 days continuously, or opioid prescriptions for 120 non-consecutive days based on previously established criteria set by CONSORT (CONsortium to Study Opioid Risks and Trends) to characterize and classify opioid use [16, 17]. Prolonged or persistent use was defined as opioid prescriptions filled 6–12 months after ASCT. High risk opioid use (> 50 MME) was defined per the CDC [5]. Opioid use was captured through recurring prescriptions in the medical record at the time of follow-up visits. Use of opioids was assessed prior to ASCT (“baseline”), during hospitalization for ASCT, at hospital discharge, and at 30-day, 90-day, 180-day, 1-year, 2-years, and 5-years post-ASCT. All patients were hospitalized for ASCT and discharged once there was hematologic recovery with adequate resolution of chemotherapy-related toxicities. The definition of new persistent use included patients who were previously opioid-naive prior to ASCT and who filled at least 3 opioid prescriptions after ASCT. Opioid data included the generic name of the opioid, the maximum daily dose prescribed, whether it was short-acting, long-acting, or both, and the documented indication. Responses were recorded with the assumption that the opioids were taken according to the prescribed dose and frequency. Daily opioid doses were converted to MME using standard conversion factors [18]. We additionally documented adjuvant analgesic (i.e., gabapentin, pregabalin, tricyclic antidepressants, or serotonin-norepinephrine reuptake inhibitors). For analysis of survival outcomes, patients were divided into two groups: chronic opioid use and non-chronic opioid use. Progression free survival (PFS) was defined as time from ASCT to progression or death. Overall survival (OS) was defined as time from ASCT to death. Survival was censored at the last outpatient clinic follow-up if the patient was still alive. Because our center is a tertiary referral center for ASCT, patient follow-up was restricted to clinic visits at our institution and not the referring practice. OS and PFS were landmarked at 6 months to remove patients who progressed before 6 months in order to determine the effect of opioid use on survival in those who had not progressed as defined by the IMWG.

### Statistical analysis

Descriptive statistics (i.e., mean and standard deviation or median and range for continuous variable, and count and percent for categorical variable) were provided for demographic, clinical, and opioid use. Chi-square tests were used for comparisons of categorical variables (i.e., to compare opioid use between patients with and without bone disease), and two-sample t-tests were used for comparisons of continuous variables. Mean MME doses received were compared between COU and non-COU patients using t-tests. All reported p-values were 2-sided, and < 0.05 were considered statistically significant. A modified Poisson regression model including factors such as age, race, presence of bone lesions, ISS, high risk FISH/karyotype, use of novel agent induction, education level, smoking, alcohol, and illicit drug use history, use of non-opioid analgesics, type of insurance, and employment status was performed to identify predictors of COU at baseline and at 6 months after ASCT. Of note, race, education level, and employment status were self-reported in the electronic health record. Landmark OS and PFS analyses were performed at 6 months after ASCT to assess the impact of ongoing COU. A multivariate Cox proportional hazards ratio model adjusted for known prognostic factors (i.e., ISS, high risk karyotype, and bony disease) was performed to identify predictors of OS.

## Results

A total of 174 patients diagnosed with MM who underwent ASCT between January 1, 2005 and December 30, 2019 were included in the final analysis. Baseline characteristics of the entire cohort are summarized in Table 1. The median age of the entire cohort was 59 (29–78) years, 90 (51.7%) of whom were male. Our patient population consisted predominantly of Black (n = 105, 60%) and Hispanic (n = 24, 13.8%) patients. At diagnosis, 111 (63.8%) patents were found to have IMWG-defined bone disease with 45 (25.9%) patients with evidence of a fracture. Most patients had either IgG subtype (n = 106, 60.9%) or light chain disease (n = 35, 20%). 48 (27.6%) patients had a high risk FISH/karyotype, and most patients were diagnosed with either ISS stage 2 (n = 45, 25.9%) or 3 (n = 54, 31%) disease. The median time to transplant was 7 (range: 3–144) months, and the median number of lines of therapy received prior to ASCT was 1 (range: 1–5). The median follow-up of our cohort was 31 (range: 1–216) months. The majority of patients received a novel agent-based induction consisting of a proteasome inhibitor or an immunomodulator drug (n = 145, 83%) and post-ASCT either single or combination maintenance therapy (n = 115, 71%).

**Table 1:** Baseline characteristics (n = 174)

Median age, range	59 (29–78)
Gender	
Male, n (%)	90 (51.7)
Female, n (%)	84 (48.3)
Race	
White, n (%)	23 (13.2)
Black, n (%)	105 (60.3)
Hispanic, n (%)	24 (13.8)
Other, n (%)	22 (12.6)
Protein Subtype	
IgG, n (%)	106 (60.9)
IgA, n (%)	33 (19)
Light Chain, n (%)	35 (20.1)
Median time to transplant, months (range)	7 (1–144)
Median number of prior lines of therapy, range	1 (1–5)
IMWG-defined bony disease	
Yes, n (%)	111 (63.8)
No, n (%)	63 (36.2)
Novel agent induction	
Yes, n (%)	145 (83.3)
No, n (%)	29 (16.7)
Post-ASCT maintenance	
Yes, n (%)	110 (67.9)
No, n (%)	52 (32.5)
High risk FISH/karyotype	
Yes, n (%)	48 (27.6)
ISS stage	
1 (n, %)	35 (20)
2 (n, %)	45 (25.9)
3 (n, %)	54 (31)

At the time of ASCT, COU was observed in 92 (52.9%) patients, 27 (29.3%) of whom did not have prior history of bone disease. The most frequently prescribed opioids were hydrocodone (n = 51, 55.4%), morphine (n = 27, 29.3%), tramadol (n = 29, 31.5%), and fentanyl (n = 10, 10.9%). Six (20.7%) patients were co-prescribed tramadol with another opioid. The baseline mean daily MME was 65.3 mg (SD = 69.97). Both COU rates (52.4% vs. 54%, p = 0.98) and average MME per day (69.5 versus 55.2 mg; p = 0.37) were similar between patients with and without bone disease. In Poisson regression analysis, previous illicit drug use was associated with a higher incidence of baseline COU (RR 4.07, 95% CI 1.53–10.82; p = 0.005). The use of non-opioid analgesic drugs (RR 0.024, 95% CI 0.09–0.63, p = 0.004), being retired (RR 0.49, 95% CI 0.29–0.83; p = 0.008), or employed (RR 0.37, 95% CI 0.15–0.91; p = 0.031) were associated with lower baseline COU. Patients with ISS stage 1 (RR 0.33, 95% CI 0.12–0.90; p = 0.03) and 2 (RR 0.25, 95% CI 0.09–0.70; p = 0.008) had a lower incidence of COU. Neither the presence of bony disease (RR 1.11, 95% CI 0.78–1.58; p = 0.569), psychiatric illness (RR 1.39, 95% CI 0.5—3.88; p = 0.526) or myeloma-related fractures (RR 1.01, 95% CT 0.68–1.51; p = 0.963) were associated with COU at the time of ASCT. Race, insurance status and education were not found to be independent predictors of baseline COU.

**Fig. 1:**
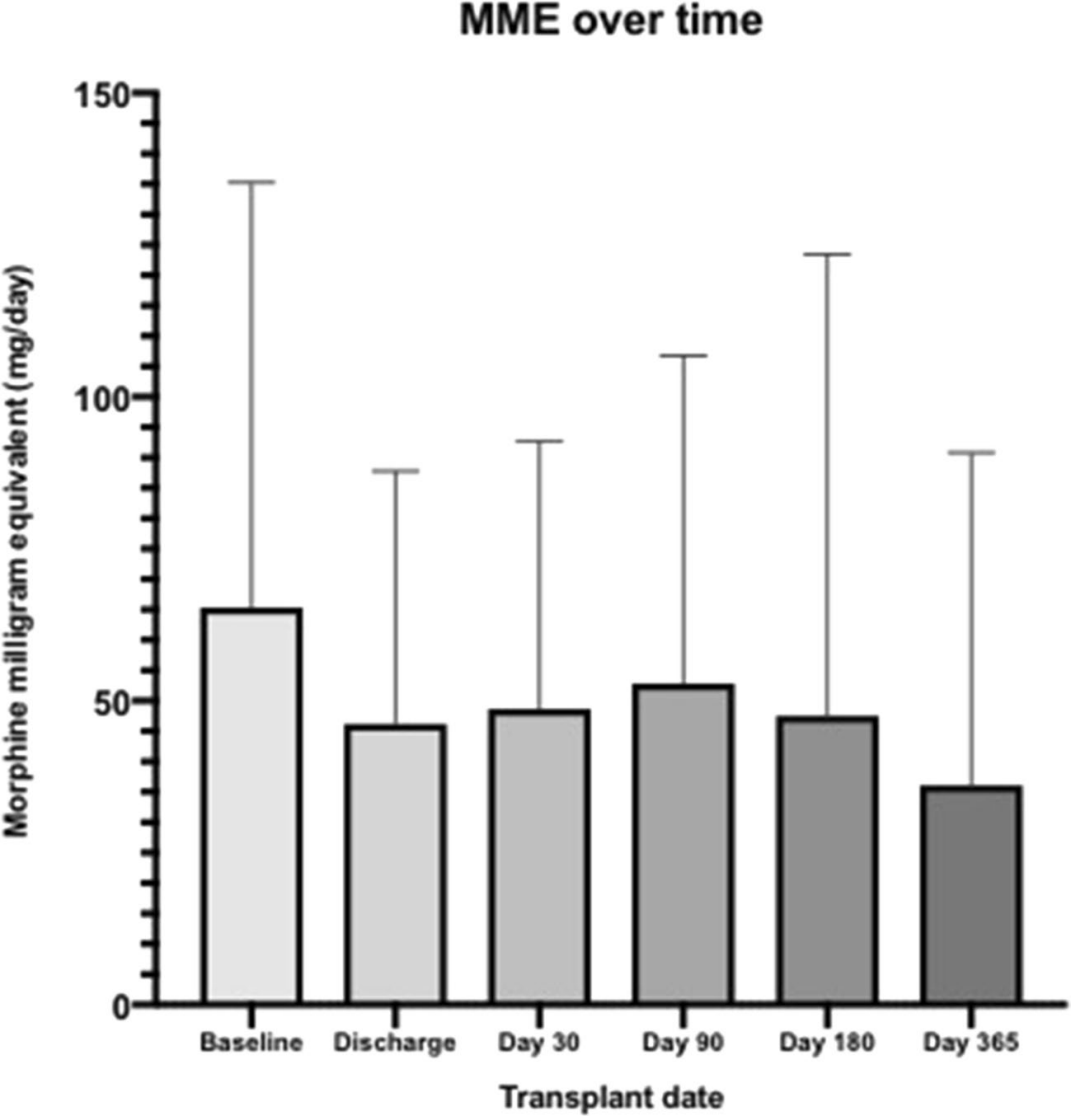
Change in MME over time. Change in daily morphine milliequivalents (MME) over time among MM patients undergoing ASCT

Among the entire cohort of 174 patients, 142 (81.6%) patients received opioids during the ASCT hospitalization, while 105 (60.3%) were discharged on opioid. Documented reasons for being discharged on opioids were the presence of musculoskeletal (n = 38, 45.8%), generalized (n = 16, 19.3%), neuropathic (n = 12, 14.5%), residual mucositis-related (n = 11, 14.5%) or headache-related (n = 6, 7.2%) pain. 72 of 174 (41.4%) patients continued to have COU at 6 months. The mean daily MME at 6 months remained relatively high at 47.15 mg (SD 74.56). The most commonly used opioids at 6 months were hydrocodone (n = 22, 30.6%), tramadol (n = 12, 16.7%), morphine (n = 7, 9.7%), and oxycodone (n = 5, 6.9%). Among the 105 patients discharged on opioids, 63 (60%) had COU at 6 months after ASCT. Furthermore, in baseline non-opioid users (n = 82), 30 (36.6%) patients became new opioid users after hospital discharge, and of these, 13 remained on opioids at 6 months. While baseline COU was not associated with higher 6-month COU, opioid use at hospital discharge was strongly associated with COU at 6 months (RR 4.21, 95% CI 1.46–12.11; p = 0.008). A trend was observed in patients with a history of psychiatric illness where they were found to be more likely to have COU at 6 months (RR 2.01, 95% CI 0.92–4.41; p = 0.081). In contrast, indicators of disease burden, including bony lesions, were not associated with 6 month COU. Figure 1 depicts daily MME patterns from baseline to 1 year after ASCT. When evaluating the change in MME from baseline to 180 days post-ASCT (with MME for new users being considered as dose escalations), we observed 23 (32%) dose escalations, 25 (34.7%) dose de-escalations, and 24 (33.3%) stable doses.

**Fig. 2:**
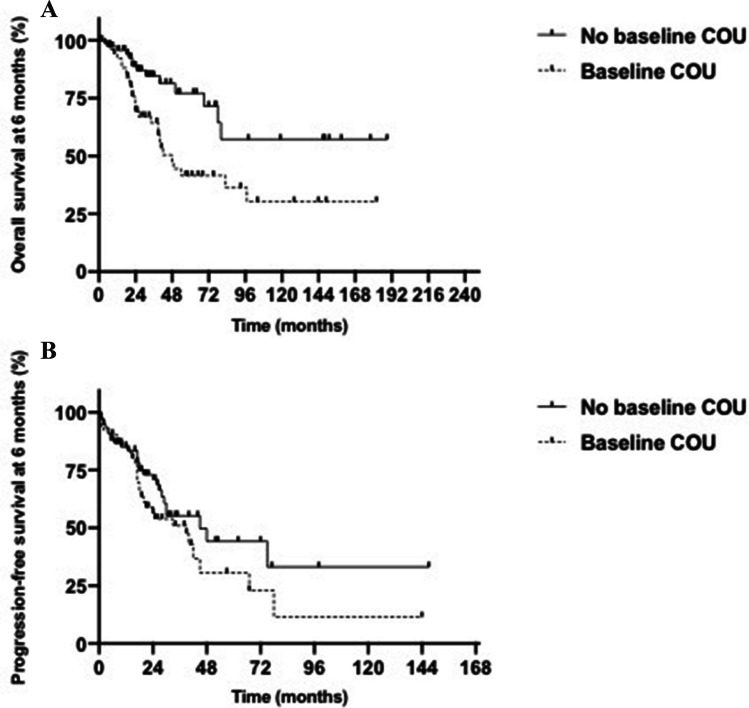
**A**. Difference in OS at 6 months between COU and non-COU users. Landmark analysis for median overall survival at 6 months in post-transplant myeloma patients in remission comparing chronic opioid use (48 months) versus no chronic opioid use (not reached) (HR 2.486, 95% CI 1.341–4.608, p = 0.004). **B.** Difference in PFS at 6 months between COU and non-COU users. Landmark analysis for median progression-free survival at 6 months in post-transplant myeloma patients in remission comparing chronic opioid use (45 months) versus no chronic opioid use (39 months) (HR 1.417, 95% CI 0.8378–2.397, p = 0.1803)

**Fig. 3:**
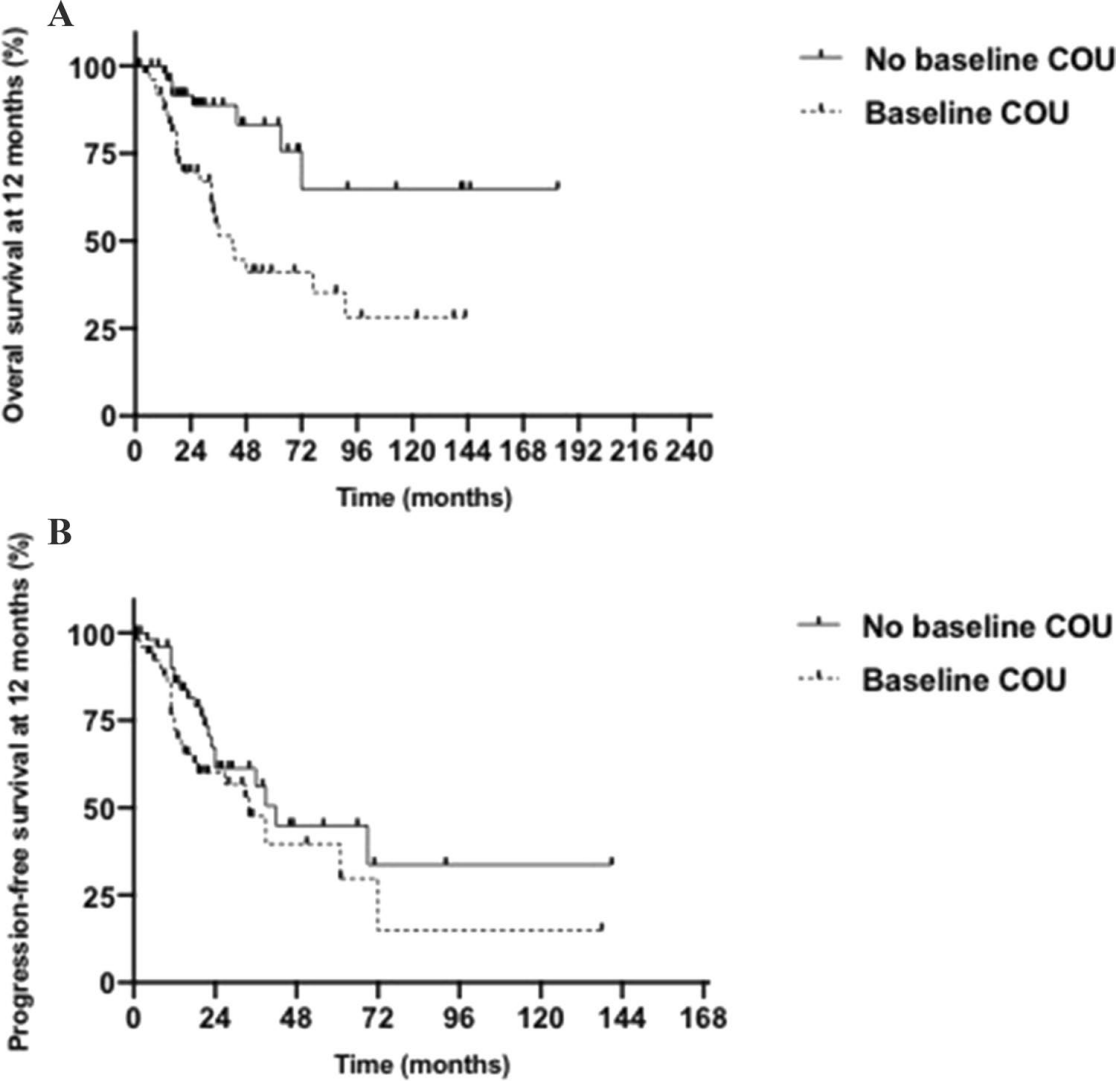
**A**. Difference in OS at 12 months between COU and non-COU users. Landmark analysis for overall survival at 12 months in post-transplant myeloma patients in remission comparing chronic opioid use (42 months) versus no chronic opioid use (not reached) (HR 3.431, 95% CI 1.733–6.794, p = 0.0011). **B.** Difference in PFS at 12 months between COU and non-COU users. Landmark analysis for progression-free survival at 12 months in post-transplant myeloma patients in remission comparing chronic opioid use (42 months) versus no chronic opioid use (34 months) (HR 1.516, 95% CI 0.8361–2.748, p = 0.1605)

For analysis of survival, patients who progressed within 6 months of ASCT were excluded from the analysis and OS and PFS was landmarked from 6 months. Although the median PFS was similar between 6-month non-COU and COU users (45 versus 39 months, p = 0.1803), OS was significantly inferior among patients with COU at 6 months (48 months versus not reached, p = 0.004; Fig. 2A and B). Similarly, in patients with COU at 1 year, PFS was similar (42 versus 34 months, p = 0.1605) but OS significantly was worse (42 months versus not reached p = 0.0011, Fig. 3A and B). In a multivariate model, we found that COU at 6 months strongly predicted for worse overall survival (HR 6.71, 95% CI 1.73–26.09; p = 0.006).

## Discussion

Pain is one of the most common and complex symptoms experienced by patients with multiple myeloma [4, 19]. While bone-directed therapies like zoledronic acid decrease skeletal-related events, we presume chronic microarchitectural changes in the bone lead to progressive deformities and persistent chronic pain. Patients can also develop painful neuropathy from the use of bortezomib, which can persist for months to years after discontinuation [20]. Additionally, as MM patients are immunosuppressed during active disease or during treatment, they can experience acute and chronic post-herpetic pain due to varicella zoster reactivation [21, 22]. Finally, during autologous transplant, patients can develop acute pain from G-CSF use or from alkylator-associated mucositis, though this follows a predictable course and resolves in most patients. Thus, opioid analgesics are often a necessary component of the multimodal treatment of acute or chronic pain in MM patients but at the same time it is well-recognized that chronic opiate use opioids at the time of transplant, it did not clarify whether this use was chronic [23]. The prevalence of COU observed at baseline in MM patients in our study is higher compared to patients with a history of cancer (42.8%; 95% CI, 41.2%−44.5%), as reported in a cross-sectional study using data from the National Survey on Drug Use and Health (NSDUH) [8]. Interestingly in our cohort, baseline COU rates were not related to the presence of bone disease as initially hypothesized but a psychosocial correlate such as previous illicit drug use was found to be a strong predictor for COU.

The dramatic improvement in therapeutic options for MM in the last two decades have led to prolonged patient survival and quality of life with a notable reduction in pain symptoms [24]. Nevertheless, given the morbidity associated with diagnosis and the prevalent use of opioids that we have shown in our population, it’s not surprising that opioid use has been shown to be more prevalent among long term MM survivors when compared to breast cancer and lymphoma survivors [25, 26]. For example, in a survey study of patients who survived 5 or more years after ASCT, MM patients reported significantly higher use of pain medications (32%) than lymphoma patients [23]. A more recent study revealed that 34.8% of MM patients were using opioids, with opioid use more frequent in younger patients and patients with bone lesions. At 1 year, 31.9% were still using opioids and continued use did not correlate with disease response. Of patients using opioids at time of ASCT, 58% either maintained or increased the dose at 1-year post-transplant [27]. Similarly, we demonstrated in our study that opioid use at hospital discharge was a strong predictor of COU at 6 months highlighting the importance to re-examine the pain regimen at each clinic visit.

As expected, myeloma patients are also not completely insulated from opioid risk, and a large study of emergency room visits for opioid overdoses among patients with cancer indicated that patients with head and neck cancer and myeloma patients were at higher risk for opioid overdose compared to patients without cancer [12]. There are limited studies of the adverse events related to opioid use in myeloma, however one qualitative study using semi-structured interviews revealed that patients recalled a median of 2 side effects related to the use of analgesics including opioids [28]. Lastly, patients with myeloma and other cancers are often vulnerable to emotional and psychological distress that might exacerbate their physical pain, increasing their likelihood of nonmedical opioid use. A study by Iqbal et al. found that among HSCT recipients, patients who were both anxious and depressed at admission had increased odds of receiving an opioid [29].

As the risks of prolonged opioid use in the absence of active disease in myeloma survivors is not known, we aimed to describe the patterns of COU among MM patients undergoing ASCT, in long term survivors, and then determine the impact of COU on survival outcomes. We demonstrated high rates of baseline COU unrelated to the presence of bone disease, and that the mean MME/day throughout the disease course is relatively high. We highlight a high rate of opiate use at discharge after ASCT, including a number of new users, which results in ongoing COU at 6 months. Exposure to opioids during treatment for acute pain (i.e., inpatient admissions) has been linked to long-term opioid use [30]. Our findings reveal the adverse effect of COU at 6 months on OS but not PFS suggesting that opioid-related deaths may be contributing to these observations. Notably, non-opioid analgesic use correlated with a lower risk of COU and thus may be appropriate in managing pain effectively and safely. We also show a high rate of co-prescribing of tramadol with other opioids. Prior studies have indicated that the highest probabilities of continued opioid use at 1 year were observed among patients who initiated treatment with a long-acting opioid followed by those whose initial treatment was with tramadol [16].

There are limitations of our study. The electronic medical record was queried to obtain active opioid prescription fill data; however, the Illinois Prescription Drug Monitoring Program (PDMP) could also have been used to capture other data including the number of prescribers and pharmacies associated with opioid prescribing. A potential risk factor for COU after ASCT is a history of opioid use preceding the diagnosis of myeloma. Due to the retrospective nature of our study, we could not accurately capture indications for pre-transplant initiation of opioids. While inpatient progress notes documented reasons for opioid prescribing during the transplant hospitalization, reasons for baseline opioid use (i.e. neuropathic versus bone pain) could not be identified reliably through the electronic health records. Therefore, it is unclear how bone and neuropathic pain trajectories changed over time and might have contributed to COU patterns. A better understanding of the type of pain being experienced will help inform tailored approaches to address specific types of pain. Despite this, we observe a spike in opioid use during hospitalization and this is worth investigating further to inform systematic interventions to improve quality of life. Furthermore, while we did not identify psychiatric illness as a predictor of COU in this study, it has been strongly linked to opioid use and misuse in cancer and noncancer patients. We relied on documented past medical histories to determine whether a patient had a psychiatric illness, but it could be that these were not formally diagnosed (i.e., anxiety, depression) or accurately documented. Race was not an independent predictor of baseline COU and we hypothesize this is likely a result of inadequate distribution of races with predominantly Black and Hispanic patients in our cohort. This warrants further investigation given that prior data has suggested differences in opioid prescribing, access to opioids, referrals to palliative care programs, and opioid overdose deaths in Black patients when compared to White patients [31–33]. Finally, given the retrospective nature of data collection, it was difficult to ascertain the cause of mortality among patients, and whether it was opioid-related, in this dataset. Our findings warrant validation using a larger dataset where data on opioid-related death is available.

In addition to frequent reassessment of the need for continued opioid use our results suggest the importance of exploring other strategies to treat pain in myeloma, including pharmacological treatments (i.e., mixed opioid agonists, methadone, topical agents, non-steroidal analgesics, THC- or CBD-based systemic or topical products), non-pharmacological therapies (i.e., physical therapy, acupuncture [34], meditation [35], exercise), neurobehavioral treatments (i.e., cognitive behavioral therapy), and interventional treatments (i.e., epidural, local nerve blocks, kyphoplasty). Additionally, during survivorship, patients often experience long term effects like anxiety, depression, pain, and effects on physical functioning [36]. Opioids may be misused to relieve these symptoms. Use of opioids to treat non-pain symptoms or to avoid the psychological distress of cancer, overlaps with opioid use disorder and is present in up to 20% of patients with cancer [37]. Thus, assessment of pain and fatigue should routinely include screening for psychological distress with interventions for psychological distress considered as adjuvant intervention strategies for pain and fatigue. Finally, clinicians should be encouraged to assess for the risk of developing OUD, with several validated screening tools available, including the Opioid Risk Tool (ORT), the Brief Risk Questionnaire (BRQ), and the Pain Medication Questionnaire (PMQ) [38–40] With increasing survival in myeloma, awareness of the risks of long-term opioid use and perhaps early involvement by palliative care, in particular during the post-transplant hospitalization phase, would enable appropriate and earlier discontinuation of opioid therapy in these patients.

## Data Availability

No datasets were generated or analysed during the current study.
